# Residual Viremia Is Linked to a Specific Immune Activation Profile in HIV-1-Infected Adults Under Efficient Antiretroviral Therapy

**DOI:** 10.3389/fimmu.2021.663843

**Published:** 2021-03-30

**Authors:** Mehwish Younas, Christina Psomas, Christelle Reynes, Renaud Cezar, Lucy Kundura, Pierre Portalès, Corinne Merle, Nadine Atoui, Céline Fernandez, Vincent Le Moing, Claudine Barbuat, Albert Sotto, Robert Sabatier, Audrey Winter, Pascale Fabbro, Thierry Vincent, Jacques Reynes, Pierre Corbeau

**Affiliations:** ^1^ Institute for Human Genetics, CNRS, Montpellier, France; ^2^ Infectious Diseases Department, Montpellier University Hospital, Montpellier, France; ^3^ Institute for Functional Genomics, Montpellier University, Montpellier, France; ^4^ Immunology Department, University Hospital, Nîmes, France; ^5^ Immunology Department, University Hospital, Montpellier, France; ^6^ IRD UMI 233, INSERM U1175, Montpellier University, Montpellier, France; ^7^ Faculty of Medicine, Montpellier University, Montpellier, France; ^8^ Infectious Diseases Department, University Hospital, Nîmes, France; ^9^ Medical Informatics Department, University Hospital, Nîmes, France

**Keywords:** low-level viremia, blip, inflammation, virologic responder, endothelium activation, coagulation

## Abstract

Chronic immune activation persists in persons living with HIV-1 even though they are aviremic under antiretroviral therapy, and fuels comorbidities. In previous studies, we have revealed that virologic responders present distinct profiles of immune activation, and that one of these profiles is related to microbial translocation. In the present work, we tested in 140 HIV-1-infected adults under efficient treatment for a mean duration of eight years whether low-level viremia might be another cause of immune activation. We observed that the frequency of viremia between 1 and 20 HIV-1 RNA copies/mL (39.5 ± 24.7% versus 21.1 ± 22.5%, p = 0.033) and transient viremia above 20 HIV-1 RNA copies/mL (15.1 ± 16.9% versus 3.3 ± 7.2%, p = 0.005) over the 2 last years was higher in patients with one profile of immune activation, Profile E, than in the other patients. Profile E, which is different from the profile related to microbial translocation with frequent CD38+ CD8+ T cells, is characterized by a high level of CD4+ T cell (cell surface expression of CD38), monocyte (plasma concentration of soluble CD14), and endothelium (plasma concentration of soluble Endothelial Protein C Receptor) activation, whereas the other profiles presented low CD4:CD8 ratio, elevated proportions of central memory CD8+ T cells or HLA-DR+ CD4+ T cells, respectively. Our data reinforce the hypothesis that various etiological factors shape the form of the immune activation in virologic responders, resulting in specific profiles. Given the type of immune activation of Profile E, a potential causal link between low-level viremia and atherosclerosis should be investigated.

## Introduction

Immune activation (IA)-related comorbidities are becoming a major concern in aviremic HIV-infected persons under antiretroviral therapy (ART) (1). For instance, atherosclerosis has been linked to inflammation and coagulation, interleukin-6 (IL-6) and D-dimer correlating positively with a greater risk of fatal cardiovascular disease (2), as well as to CD8+ T cell activation (HLA-DR and CD38 coexpression) (3) and monocyte (soluble CD163 level and cell surface coexpression of CD14 and CD16) activation (3, 4). It is therefore important to better identify the causes of this IA. HIV itself may be one of these drivers. Although HIV RNA levels are below the detection level of routine tests in these patients, most of them still produce low levels of virus, and HIV (glyco)proteins may be detected in their lymph nodes (5). Many HIV components may directly activate the innate immune system. For example, gp120 (6) Nef and Vpr (7) have been reported to directly activate monocytes and macrophages, and gp41 T cells (8). It has also been reported that HIV RNA induces interferon-α production by plasmacytoid dendritic cells *via* TLR-7 and TLR-9. Moreover, HIV antigens detected by specific B lymphocytes and T cells trigger an adaptive immune response. Finally, even a truncated CD4+ T cell infection may induce caspase-1 activation and IL-1β production in CD4+ T cells (9). In addition, HIV might also favor other causes of IA (10). Thus, Nef protein is also known to interact with the adenosine-triphosphate-binding cassette A1 transporter. This interaction interferes with cholesterol metabolism, and may promote pro-inflammatory metabolic disorders (11). Likewise, HIV-mediated IA might promote immune senescence, which is an additional cause of IA.

Yet authors looking for correlations between residual viremia and IA markers obtained contradictory results. Some authors found a link between residual viremia and inflammation (12), CD4+ T cell activation (13), and monocyte activation (12). By contrast, other authors did not observe such correlations (14–17).

Recent data however argue for a role of persistent viral production in IA. First, in virologic responders, higher levels of the inflammatory markers C-reactive protein (CRP), Tumor Necrosis Factor α (TNFα), IL-6, interferon γ (IFNγ) and/or the monocyte activation marker soluble CD14 (sCD14) have been reported in non-fully adherent patients than in fully adherent patients (18, 19). Second, in elite controllers, who are aviremic in the absence of treatment, intermittent bouts of viremia, so-called blips, are associated with a low CD4/CD8 ratio which is a global marker of IA (20). Moreover, the initiation of ART in these patients reduces CD4+ T cell and CD8+ T cell activation in the blood as well as in the gastrointestinal associated lymphoid tissues (21). Third, switching from a 3-drug regimen to some 2-drug ART regimens may result in an increase in CD8+ T cell counts (22, 23).

Knowing whether low-level viremia is a cause of IA is particularly important at a time when 2-drug regimens rather than 3-drug ART regimens (24), day-on, day-off schedules (25), and intermittent four-days-a-week treatments (26) are being proposed.

In a previous study, we analyzed 64 soluble and cell surface markers of inflammation and CD4+ and CD8+ T cell, B cell, monocyte, NK cell, and endothelial activation in 140 adults under effective ART. A double hierarchical clustering of patients and markers unveiled that these virologic responders had 5 different IA profiles (27). The first profile was characterized by a high percentage of central memory CD8+ T cells, the second by a low CD4:CD8 ratio, the third by frequent HLA-DR+ CD4+ T cells, and the two last profiles by an elevated proportion of CD38-expressing CD8+ and CD4+ T cells, respectively. In this present study, we looked for a link between one of those profiles and residual viremia.

## Materials and Methods

### Study Design

This cross-sectional observational study has already been previously described (27). We recruited 140 HIV-1-infected adults with CD4 count above 200 cells/µL. Viremia of all participants, under stable ART, was below 50 copies per mL for at least 6 months before inclusion. We also recruited 150 persons with a mean ± SD age of 62 ± 4 years in the general population. Pregnant or breastfeeding women, persons under treatment or presenting a disease likely to modify the immune system were not included. Blood samples for immune profiling were collected once between April 2014 and June 2016. This study was approved by the Ethics Committee of Montpellier University Hospital. All patients provided written informed consent. The trial was registered on ClinicalTrials.gov (NCT02334943).

### Immune Activation Markers

Using flow cytometry as previously described (28), we determined cell surface markers for the differentiation, activation, senescence, and/or exhaustion of CD4+ T lymphocytes, CD8+ T lymphocytes, and NK cells. We used ELISA to quantify soluble TNF receptor I (sTNFRI), sCD14 and soluble CD163 (sCD163), tissue Plasminogen Activator (tPA), soluble Thrombomodulin (sTM), and soluble Endothelial Protein C Receptor (sEPCR) and, by turbidimetry, CRP, immunoglobulins (Ig) and D-dimers also as previously reported (28).

### Residual Viremia

HIV-1 RNA plasma level was quantified using the Amplicor HIV-1 Monitor test (Roche Diagnostic Systems). The test has a positive threshold of 20 copies/mL, and discriminates samples containing no detectable copies in 1mL from samples containing 1 to 20 copies/mL. We also recorded the percentage of blips, defined as isolated episodes of viremia > 20 copies/mL, within the 2 years preceding the analysis of the immune profile. The frequency of detectable viremia and blips over the 2 last years was determined on 4 to 8 measurements.

### Statistical Analysis

Fisher’s exact test or χ2 test was used to compare qualitative covariates. For quantitative covariates, on one hand, when comparing two groups (e.g. undetectable vs detectable viremia), we compared the distributions of covariates using the Student T-test or the Wilcoxon-Mann-Whitney test as appropriate. On the other hand, when comparing profiles together for example (i.e. more than two groups), the comparison was made using Anova or the Kruskal-Wallis H test as appropriate. Correlations were evaluated by Pearson, Spearman or Kendall test as appropriate. Normality was assessed using the Shapiro-Wilk test.

All analyses were performed using R software, version 3.6.1 (R Development Core Team, A Language and Environment for Statistical Computing, Vienna, Austria, 2016. https://www.R-project.org/).

### Funding Source

Nîmes and Montpellier University Hospitals, MSD and MSDAVENIR.

## Results

### Study Subjects

HIV-1-infected adults (n = 140), under efficient ART for a mean (SD) duration of 7.9 (4.1) years, were recruited at the University Hospitals of Nîmes and Montpellier, France. Eighty-one percent of patients were male, with a mean age of 56 (9) years. Their pretherapeutic CD4 count and viremia were 199 (119) cells/µL and 1,4372,216 (9,602,969) HIV RHA copies/mL, respectively. Their current mean CD4 count was 733 ± 375 cells/µL, and their CD4/CD8 ratio 1.24 (0.88). ART regimens and co-infections are given in **Table 1**.

**Table 1:** Bioclinical characteristics of the study population.

Characteristics		HIV+ Treated
Number of individuals		140
Nucleoside reverse transcriptase inhibitor	N (%)	128 (91)
Non-nucleoside reverse transcriptase inhibitor	N (%)	50 (36)
Protease inhibitor	N (%)	67 (48)
Integrase inhibitor	N (%)	44 (31)
HBs Ag+	N (%)	3 (2)
Anti-HBs Ab+	N (%)	61 (44)
Anti-HBc Ab+	N (%)	54 (39)
HCV coinfection	N (%)	6 (4)
CMV coinfection	N (%)	124 (89)
EBV coinfection	N (%)	138 (99)
HAV coinfection	N (%)	97 (69)

HBs Ag, Hepatitis B surface antigen; HBc, Hepatitis B core; HCV, Hepatitis C Virus; CMV, Cytomegalovirus; EBV, Epstein-Barr Virus; HAV, Hepatitis A Virus.

### Residual Viremia

In this study population, the frequency of residual viremia (1-20 copies/mL) at the time of study was 26%. There was no difference, neither in age (56.1 ± 8.5 versus 55.9 ± 10.1 years, p = 0.771), duration of infection (14.5 ± 8.0 versus 16.8 ± 7.7 years, p = 0.168) nor aviremia (7.0 ± 3.5 versus 8.3 ± 4.2 years, p = 0.155), nor in pretherapeutic CD4 count (164 ± 110 versus 159 ± 103 cells/mL, p = 0.980) nor pretherapeutic viremia (4,782,630 ± 20,381,332 versus 530,006 ± 1,733,809 copies/mL, p = 0.831) between patients with detectable and undetectable viremia, respectively. Likewise, no link could be established between residual viremia and the use of nucleoside reverse transcriptase inhibitors (p = 0.999), non-nucleoside reverse transcriptase inhibitors (p = 0.999), protease inhibitors (p = 0.969), or integrase inhibitors (p = 0.900).

**Figure 1:**
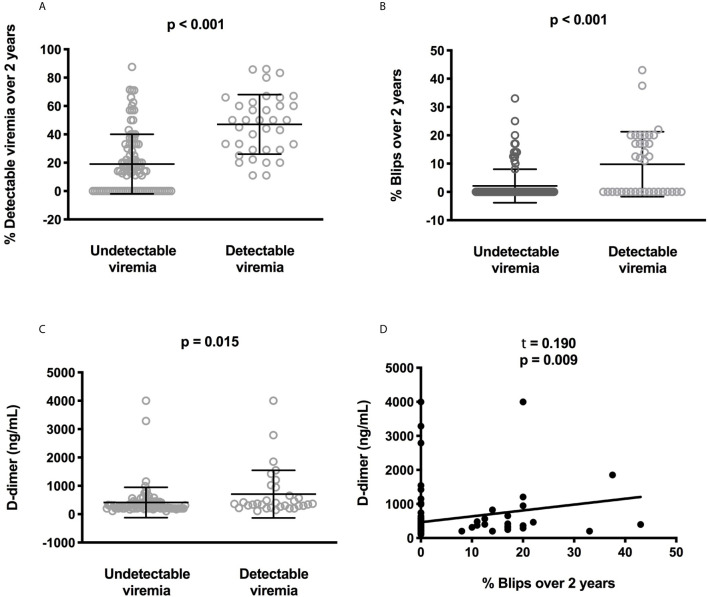
Correlations between low-level viremia, blips, and D-dimer plasma levels. Difference for each participant in the frequency where the participant displayed low-level viremia **(A)** or blips **(B)** over the two last years between participants with or without current detectable viremia. Difference in circulating D-dimer concentrations between participants with or without current detectable viremia. Statistical analyses were performed using a Mann-Whitney test **(C)**. Correlation between the frequency of blips over the two last years and D-dimer plasma levels. Statistical analysis was performed using Kendall correlation **(D)**.

The percentage of samples with detectable viremia (1-20 HIV-1 RNA copies/mL) or blips (isolated viremia > 20 HIV-1 RNA copies/mL) during the two years preceding the study was available for 135 out of 140 participants. These percentages of samples with detectable viremia (47.1 ± 21.0% versus 19.0 ± 21.0%, p < 10^-4^, **Figure 1A**) and blips (9.8 ± 11.5% versus 2.1 ± 5.9%, p < 10^-4^, **Figure 1B**) were higher in patients with detectable viremia than in patients with undetectable viremia at the time of the study.

### Relationships Between Residual Viremia and Biomarkers

We determined the numbers and percentages of the following subpopulations: (i) activated (HLA-DR+ and/or CD38+), exhausted (PD-1+), senescent (CD57+, eventually CD27- and CD28-), naïve (CD45RA+CD27+), central and effector (CD45RA-CD27+ and CD45RA-CD27-, respectively) memory CD4+ and CD8+ T cells, (ii) activated (HLA-DR+), dysfunctional (CD56-), and senescent (CD57+) NK cells. Monocyte activation was evaluated by measuring sCD14 and sCD163, and B cell activation by measuring IgM, IgG, and IgA levels. Inflammation was monitored by quantifying sTNFRI and CRP. tPA, sTM, and sEPCR were used as markers of endothelium activation, and D-dimers were used as an indicator of fibrinolysis.

**Figure 2:**
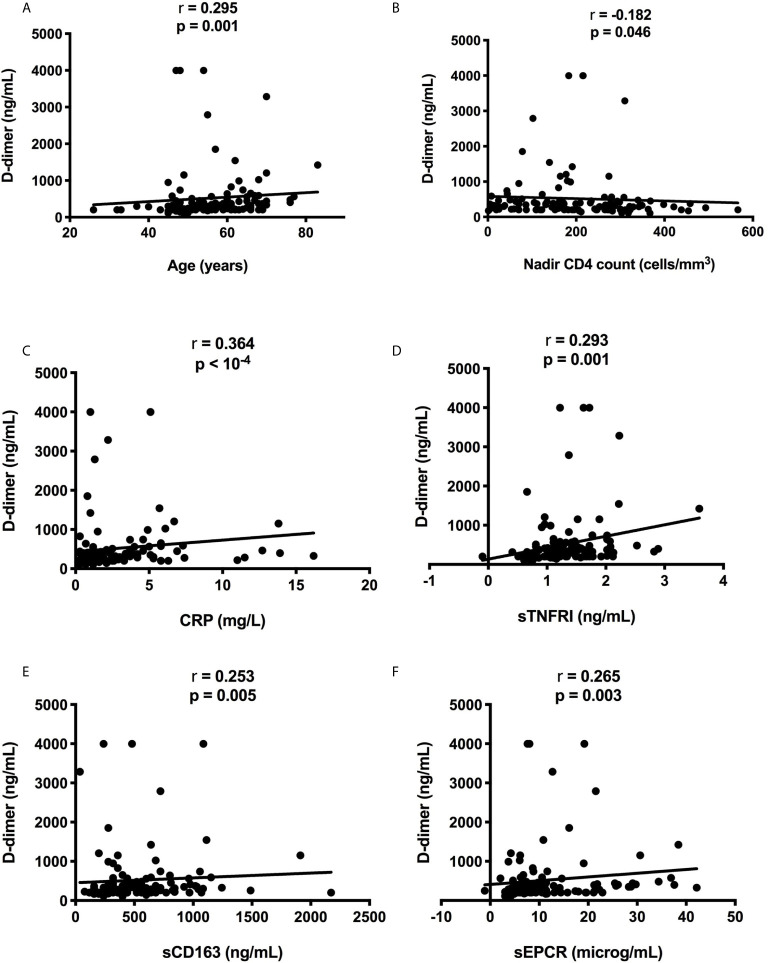
Correlations between D-dimer level, participant age **(A)** and pretherapeutic CD4 count **(B)**, CRP **(C)**, sTNFRI **(D)**, sCD163 **(E)**, and sEPCR **(F)**.

We compared residual viremia and blip frequency, i.e. for each participant, the frequency where the participant displayed low-level viremia or blips during the two last years with each one of these markers. The only link we revealed was with D-dimers. Patients with 1-20 HIV-1 RNA copies at the time of the study presented higher D-dimer levels than patients with undetectable viremia (n = 119, 708 ± 841 versus 411 ± 535 ng/mL, p = 0.015, **Figure 1C**). Moreover, there was a correlation between blip frequency over the two last years and D-dimer levels (n = 119, Kendall coefficient = 0.190, p = 0.009, **Figure 1D**). Looking for correlations between D-dimers and patient characteristics as well as other markers, we observed links between the coagulation marker, participant age (r = 0.295, p = 0.001, **Figure 2A**), pretherapeutic CD4 count (r = -0.182, p = 0.046, **Figure 2B**), the inflammation markers CRP (r = 0.364, p < 10^-4^, **Figure 2C**) and sTNFRI (r = 0.293, p = 0.001, **Figure 2D**), the monocyte activation marker sCD163 (r = 0.253, p = 0.005, **Figure 2E**), and the endothelial activation marker sEPCR (r = 0.265, p = 0.003, **Figure 2F**).

### Residual Viremia Over Time Is Related to a Specific Immune Activation Profile

We previously reported that two independent hierarchical clustering analyses of the activation markers for the 140 patients had identified 5 groups of individuals presenting different IA profiles (Profiles A to E). Duration of infection and aviremia, nadir CD4, pretherapeutic viral load and CD4:CD8 ratio, as well as age were not different between Profile E and the other Profiles (27). There was differences neither in nucleoside reverse transcriptase inhibitor (p = 0.565), non-nucleoside reverse transcriptase inhibitor (p = 0.999), protease inhibitor (p = 0.165), nor in integrase inhibitor (0.999) usage between Profile E and the other profiles. The only difference was a tendency to a higher proportion of females in Profile E (p = 0. 061) (27). Yet, the frequency of detectable viremia (p = 0.999) or blips (p = 0.278) during the two years were not different between males and females (data not shown).

**Figure 3:**
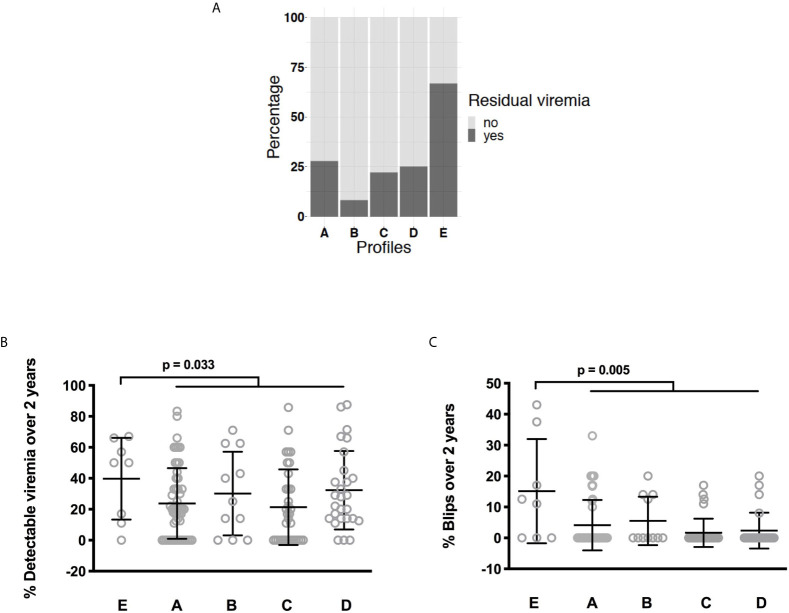
Differences in the frequency of current low-level viremia between the various IA profiles. Statistical analyses were performed using a Kruskal-Wallis H test **(A)**. Differences in the frequency of low-level viremia **(B)** and blips **(C)** over the two last years between the various IA profiles. Statistical analyses were performed using a Mann-Whitney test.

Interestingly, one of these profiles, Profile D, was linked to microbial translocation (27). We reasoned that another IA profile could be linked to another cause of IA, residual viral production. To test this hypothesis, we compared the frequency of residual viremia and the occurrence of blips over time between the IA profiles. Detectable viremia at the time of the study was more frequent in patients with Profile E than in the other patients (66.7% versus 23.8%, p = 0.011, **Figure 3A**). The frequency of detectable viremia over the 2 last years was also higher in Profile E patients than in patients with other profiles (39.5 ± 24.7% versus 21.1 ± 22.5%, p = 0.033, **Figure 3B**). Likewise, previous blip frequency was higher in Profile E patients than in the patients with other profiles (15.1 ± 16.9% versus 3.3 ± 7.2%, p = 0.005, **Figure 3C**).

### Characterization of the Immune Activation Profile Related to Residual Viremia

**Figure 4:**
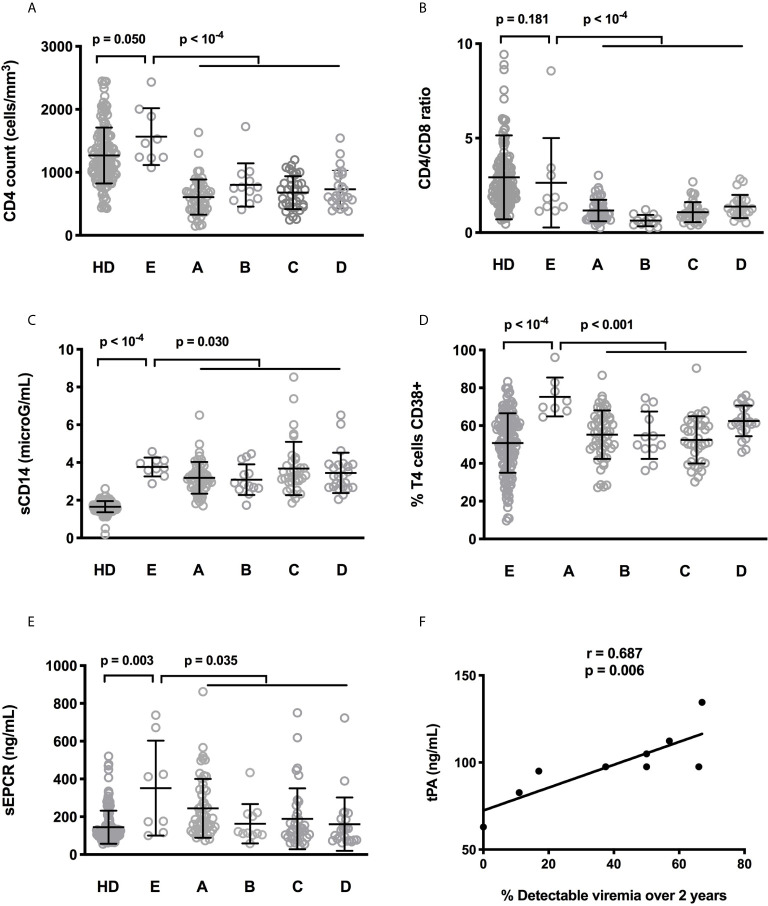
Differences in CD4 count **(A)**, CD4:CD8 ratio **(B)**, sCD14 **(C)**, frequency of CD4+ T cells expressing CD38 **(D)**, and sEPCR **(E)** between healthy donors (HD) and people living with HIV-1 with the five IA profiles. Statistical analyses were performed using a Mann-Whitney test. Correlation between low-level viremia over the two last years and tPA in Profile E participants. Statistical analysis was performed using Spearman correlation **(F)**.

No difference in duration of infection (p = 0.191) and of viral suppression (p = 0.285), in pretherapeutic viremia (p = 0.881) or CD4 count (0.285), and in age (p = 0.138) was observed between Profile E patients and patients with other profiles (data not shown). Compared with the other patients, Profile E patients had higher CD4 counts (1567 ± 451 versus 669 ± 288 cells/mL, p < 10^-4^, **Figure 4A**) and CD4:CD8 ratios (2.79 ± 2.48 versus 1.14 ± 0.57, p < 0.001, **Figure 4B**). They also presented higher levels of sCD14 (3.76 ± 0.50 versus 3.37 ± 1.08 mg/mL, p = 0.030, **Figure 4C**) and higher percentages of CD38-positive CD4+ T-cells (73.1 ± 11.4% versus 55.9 ± 12.4%, p < 10^-4^, **Figure 4D**) compared with the other patients. Profile E was also characterized by a high level of the endothelium activation marker sEPCR (18 ± 12% versus 10 ± 8%, p = 0.035, **Figure 4E**). Moreover, in Profile E patients there was a strong link between frequency of detectable viremia over the 2 last years and another endothelium activation marker, tPA (r = 0.687, p = 0.006, **Figure 4F**).

## Discussion

It is logical to assume that residual viremia should fuel immune activation in virological responders. Yet, although some authors have indeed found a link between low-level viremia and IA (12, 13), others have not (14–17). This discrepancy might be due to differences in the size and/or bioclinical characteristics of the populations under study, as well as the actual definition of residual viremia. Here, we show that the frequency of detectable HIV-1 RNA and blips over a period of 2 years is associated with a specific profile of IA, Profile E. There may be two explanations for why we observed such a correlation whereas other authors had failed to identify it. First, a 24-month follow-up of viral load is more sensitive than a single measurement. Second, our two-step approach, consisting of clustering IA profiles in the patient population and thereafter looking for correlations between each of these profiles and residual viremia, may have unveiled links which would have been hidden in searches for such links in the global population. For instance, in our study, whereas high sCD14 levels and percentages of CD4+ T cells expressing CD38 characterize Profile E, these markers are not associated with residual viremia when taking into account the 140 participants.

The high circulating concentrations of sCD14 that we observed in Profile E patients is in line with the link between this IA marker and residual viremia reported by other authors (12). This link might be explained by the fact that the presence of viral components within the gut-associated lymphoid tissues might be responsible for local inflammation and thereby an increase in gut permeability facilitating microbial translocation (29). This could thus be a first argument in favor of a causal link between residual viremia and Profile E.

Previously, CD4+ T cell activation has also been found to be associated with residual viremia (13). Here again, it may be the presence of viral components that trigger this form of IA. Indeed, gp41 has been reported to interact with the T cell receptor and to facilitate thereby T lymphocyte activation (8). Moreover, Doitsh et al. observed that the presence of HIV DNA in CD4+ T cell provokes inflammasome activation, even though the viral life cycle is truncated (9). Consequently, CD4+ T cell activation in Profile E patients may be induced by the presence of the virus.

Compared with the other IA profiles, Profile E is also characterized by a high level of sEPCR. sEPCR results from the cleavage of the external portion of the protein C receptor on the surface of endothelial cells activated by thrombin, TNFα, IL-1 or endotoxins (30). An increase in sEPCR has already been reported in HIV infection (31, 32). A decrease in sEPCR under 48-week ART has even been correlated with HIV RNA changes (32). This is in line with the hypothesis that the presence of HIV may provoke EPCR cleavage, and further argues for a model in which Profile E is the consequence of residual viral production.

One consequence of our results is that we now know that residual viremia should be sought in patients with increased proportions of CD38+CD4+ T cell, sCD14 and/or sEPCR. Moreover, in these patients, potential causes of this residual viremia, e.g., non-optimal adherence to treatment or partial resistance to the current antiretroviral regimen, should also be investigated.

Globally, the present data support the idea that, depending on the etiological factor at work, microbial translocation for Profile D patients (27) and residual viremia for Profile E patients (present study), virologic responders develop a particular IA profile. As we have also established that a specific IA profile may be related to a specific comorbidity, insulin resistance (28), we propose a model in which a cause of IA may fuel an IA profile that may favor an IA-related morbidity. To further test this hypothesis, tissue evaluation of the IA, particularly in secondary lymphoid organs, and functional assays would be necessary. According to this model, the fact that we have shown that residual viremia is associated with a marker of monocyte activation (sCD14), two markers of endothelial activation (sEPCR and tPA), and a marker of coagulation (D-dimer), three forms of activation known to favor atherothrombosis (33–35), should encourage us to search for a link between residual viremia and atherosclerosis.

## Data Availability Statement

The raw data supporting the conclusions of this article will be made available by the authors, without undue reservation.

## Ethics Statement

The studies involving human participants were reviewed and approved by Ethics Committee of Montpellier University Hospital, France. The patients/participants provided their written informed consent to participate in this study.

## Author Contributions

MY and LK contributed to the design of the flow cytometry study, and acquired, analyzed, and interpreted cell surface and soluble marker data. CP contributed to the design of the study, the enrolment of patients, and acquired, analyzed and interpreted the data. RC, PP, and TV contributed to the design of the flow cytometry study and acquired, analyzed and interpreted cell surface markers. CR, RS, AW, and PF contributed to the design of the statistical study and acquired, analyzed, and interpreted the statistical data. CM, NA, CF, VL, CB, and AS acquired, analyzed, and interpreted clinical data. JR contributed to the design of the study and analyzed and interpreted the data. PC contributed to the design of the study, analyzed, and interpreted data, and wrote the first draft of the manuscript. All authors contributed to the article and approved the submitted version.

## Funding 

The study was funded by MSD, MSDAvenir, and the university hospitals of Montpellier and Nîmes. These sponsors had no role in the study design, the collection, analysis, or interpretation of data, the writing of the report, nor in the decision to submit the paper for publication.

## Conflict of Interest

The authors declare that the research was conducted in the absence of any commercial or financial relationships that could be construed as a potential conflict of interest.

## References

[1] Miller CJ Baker JV Bormann AM Erlandson KM Hullsiek KH Justice AC. Adjudicated morbidity and mortality outcomes by age among individuals with HIV infection on suppressive antiretroviral therapy. PLoS One (2014) 9:e95061. 10.1371/journal.pone.0095061 24728071 PMC3984283

[2] Nordell AD McKenna M Borges AH Duprez D Neuhaus J Neaton JD. Severity of cardiovascular disease outcomes among patients with HIV is related to markers of inflammation and coagulation. J Am Heart Assoc (2014) 3:e000844. 10.1161/JAHA.114.000844 24870935 PMC4309077

[3] Longenecker CT Funderburg NT Jiang Y Debanne S Storer N Labbato DE. Markers of inflammation and CD8 T-cell activation, but not monocyte activation, are associated with subclinical carotid artery disease in HIV-infected individuals. HIV Med (2013) 14:385–90. 10.1111/hiv.12013 PMC3640592 23332012

[4] Fitch KV Srinivasa S Abbara S Burdo TH Williams KC Eneh P. Noncalcified coronary atherosclerotic plaque and immune activation in HIV-infected women. J Infect Dis (2013) 208:1737–46. 10.1093/infdis/jit508 PMC3814845 24041790

[5] Popovic M Tenner-Racz K Pelser C Stellbrink H-J van Lunzen J Lewis G. Persistence of HIV-1 structural proteins and glycoproteins in lymph nodes of patients under highly active antiretroviral therapy. Proc Natl Acad Sci U S A (2004) 102:14807–12. 10.1073/pnas.0506857102 PMC1253583 16199516

[6] Planès R Serrero M Leghmari K BenMohamed L Bahraoui B. HIV-1 Envelope Glycoproteins Induce the Production of TNF-α and IL-10 in Human Monocytes by Activating Calcium Pathway. Sci Rep Sci Rep (2018) 8(1):17215. 10.1038/s41598-018-35478-1 30464243 PMC6249280

[7] Lawn SD Butera ST Folks TM. Contribution of immune activation to the pathogenesis and transmission of human immunodeficiency virus type 1 infection. Clin Microbiol (2001) 14:753–77. 10.1128/CMR.14.4.753-777.2001 PMC89002 11585784

[8] Yakovian O Schwarzer R Sajman J Neve-Oz Y Razvag Y Herrmann A. Gp41 dynamically interacts with the TCR in the immune synapse and promotes early T cell activation. Sci Rep (2018) 8:9747. 10.1038/s41598-018-28114-5 29950577 PMC6021400

[9] Doitsh G Cavrois M Lassen KG Zepeda O Yang Z Santiago ML. Abortive HIV infection mediates CD4 T cell depletion and inflammation in human lymphoid tissue. Cell (2010) 143:789–801. 10.1016/j.cell.2010.11.001 21111238 PMC3026834

[10] Younas M Psomas C Reynes J Corbeau P. Immune activation in the course of HIV-1 infection: Causes, phenotypes and persistence under therapy. HIV Med (2016) 17:89–105. 10.1111/hiv.12310 26452565

[11] Mujawar Z Rose H Morrow MP Pushkarsky T Dubrovsky L Mukhamedova N. Human immunodeficiency virus impairs reverse cholesterol transport from macrophages. PLoS Biol (2006) 4:e365. 10.1371/journal.pbio.0040365 17076584 PMC1629034

[12] Falasca F Di Carlo D De Vito C Bon I d’Ettorre G Fantauzzi A. Evaluation of HIV-DNA and inflammatory markers in HIV-infected individuals with different viral load patterns. BMC Infect Dis (2017) 17:581. 10.1186/s12879-017-2676-2 28830393 PMC5568129

[13] Hatano H Jain V Hunt PW Lee TH Sinclair E Do TD. Cell-based measures of viral persistence are associated with immune activation and programmed cell death protein 1 (PD-1)-expressing CD4+ T cells. J Infect Dis (2013) 213:370–8. 10.1093/infdis/jis630 PMC3666131 23089590

[14] Guihot A Dentone C Assoumou L Parizot C Calin R Seang S. Residual immune activation in combined antiretroviral therapy-treated patients with maximally suppressed viremia. AIDS (2016) 30:327–30. 10.1097/QAD.0000000000000815 26186129

[15] Gandhi RT McMahon DK Bosch RJ Lalama CM Cyktor JC Macatangay BJ. Levels of HIV-1 persistence on antiretroviral therapy are not associated with markers of inflammation or activation. PLoS Pathog (2011) 13:e1006285. 10.1371/journal.ppat.1006285 PMC5398724 28426825

[16] Chun T-W Murray D Justement JS Hallahan CW Moir S Kovacs C. Relationship between residual plasma viremia and the size of HIV proviral DNA reservoirs in infected individuals receiving effective antiretroviral therapy. J Infect Dis (2011) 204:135–8. 10.1093/infdis/jir208 PMC3105034 21628667

[17] Allavena C Rodallec A Sécher S Reliquet V Baffoin S André-Garnier E. Evaluation of residual viremia and quantitation of soluble CD14 in a large cohort of HIV-infected adults on a long-term non-nucleoside reverse transcriptase inhibitor-based regimen. J Med Virol (2013) 85:1878–82. 10.1002/jmv.23679 23861166

[18] Castillo-Mancilla JR Brown TT Erlandson KM Palella FJJ Gardner EM Macatangay BJC. Suboptimal Adherence to Combination Antiretroviral Therapy Is Associated With Higher Levels of Inflammation Despite HIV Suppression. Clin Infect Dis (2016) 63:1661–7. 10.1093/cid/ciw650 PMC5146724 27660234

[19] Castillo-Mancilla JR Morrow M Boum Y Byakwaga H Haberer JE Martin JN. Brief Report: Higher ART Adherence Is Associated With Lower Systemic Inflammation in Treatment-Naive Ugandans Who Achieve Virologic Suppression. J Acquir Immune Defic Syndr (2018) 77:507–13. 10.1097/QAI.0000000000001629 PMC5844840 29346185

[20] Chereau F Madec Y Sabin C Obel N Ruiz-Mateos E Chrysos G. Impact of CD4 and CD8 dynamics and viral rebounds on loss of virological control in HIV controllers. PLoS One (2017) 12:e0173893. 10.1371/journal.pone.0173893 28380038 PMC5381858

[21] Hatano H Yukl SA Ferre AL Graf EH Somsouk M Sinclair E. Prospective antiretroviral treatment of asymptomatic, HIV-1 infected controllers. PLoS Pathog (2013) 9:e1003691. 10.1371/journal.ppat.1003691 24130489 PMC3795031

[22] Mussini C Lorenzini P Cozzi-Lepri A Marchetti G Rusconi S Gori A. Switching to dual/monotherapy determines an increase in CD8+ in HIV-infected individuals: an observational cohort study. BMC Med (2018) 16:79. 10.1186/s12916-018-1046-2 29807541 PMC5972434

[23] Quiros-Roldan E Magro P Raffetti E Izzo I Borghetti A Lombardi F. Biochemical and inflammatory modifications after switching to dual antiretroviral therapy in HIV-infected patients in Italy: a multicenter retrospective cohort study from 2007 to 2015. BMC Infect Dis (2018) 18 285. 10.1186/s12879-018-3198-2 PMC6020212 29940869

[24] Moreno S Perno CF Mallon PW Behrens G Corbeau P Routy JP. Two-drug vs. three-drug combinations for HIV-1: Do we have enough data to make the switch? HIV Med (2019) 20(Suppl 4):2–12. 10.1111/hiv.12716 30821898

[25] Costantini A Tontini C Rocchi M Martini M Butini L. Day-On, Day-Off emtricitabine, tenofovir disoproxil fumarate and efavirenz single tablet regimen (DODO) as maintenance therapy in HIV-infected patients. Infez Med (2018) 26:126–32. 29932084

[26] de Truchis P Assoumou L Landman R Mathez D Le Dû D Bellet J. Four-days-a-week antiretroviral maintenance therapy in virologically controlled HIV-1-infected adults: the ANRS 162-4D trial. J Antimicrob Chemother (2018) 73:738–47. 10.1093/jac/dkx434 29186458

[27] Younas M Psomas C Reynes C Cezar R Kundura L Portales P. Microbial Translocation Is Linked to a Specific Immune Activation Profile in HIV-1-Infected Adults With Suppressed Viremia. Front Immunol (2019) 10:2185. 10.3389/fimmu.2019.02185 31572392 PMC6753629

[28] Psomas C Younas M Reynes C Cezar R Portales P Tuaillon E. One of the immune activation profiles observed in HIV-1-infected adults with suppressed viremia is linked to metabolic syndrome: The ACTIVIH study. EBioMedicine (2016) 8:265–76. 10.1016/j.ebiom.2016.05.008 PMC4919610 27428436

[29] Reus S Portilla J Sánchez-Payá J Giner L Francés R Such J. Low-level HIV viremia is associated with microbial translocation and inflammation. J Acquir Immune Defic Syndr (2013) 62:129–34. 10.1097/QAI.0b013e3182745ab0 23018379

[30] Van de Wouwer M Collen D Conway EM. Thrombomodulin-protein C-EPCR system: integrated to regulate coagulation and inflammation. Arterioscler Thromb Vasc Biol (2004) 24:1374–83. 10.1161/01.ATV.0000134298.25489.92 15178554

[31] Nozza S Pogliaghi M Chiappetta S Spagnuolo V Fontana G Razzari C. Levels of soluble endothelial protein C receptor are associated with CD4+ changes in Maraviroc-treated HIV-infected patients. PLoS One (2012) 7:e37032. 10.1371/journal.pone.0037032 22715361 PMC3371054

[32] Chiappetta S Ripa M Galli L Razzari C Longo V Galli A. Soluble endothelial protein C receptor (sEPCR) as an inflammatory biomarker in naive HIV-infected patients during ART. J Antimicrob Chemother (2016) 71:1627–31. 10.1093/jac/dkw010 26888911

[33] Reynolds HR Buyon J Kim M Rivera TL Izmirly P Tunick P. Association of plasma soluble E-selectin and adiponectin with carotid plaque in patients with systemic lupus erythematosus. Atherosclerosis (2010) 210:569–74. 10.1016/j.atherosclerosis.2009.12.007 PMC3963602 20044088

[34] Masiá M Padilla S García JA García-Abellán J Fernández M Bernardino I. Evolving understanding of cardiovascular, cerebrovascular and peripheral arterial disease in people living with HIV and role of novel biomarkers. A study of the Spanish CoRIS cohort, 2004-2015. PLoS One (2019) 14:e0215507. 10.1371/journal.pone.0215507 31026289 PMC6485642

[35] Engelberger RP Limacher A Kucher N Baumann F Silbernagel G Benghozi R. Biological variation of established and novel biomarkers for atherosclerosis: Results from a prospective, parallel-group cohort study. Clin Chim Acta (2015) 447:16–22. 10.1016/j.cca.2015.05.003 25979692

